# Supplementary material for: Mapping Geographical Differences and Examining the Determinants of Childhood Stunting in Ethiopia: A Bayesian Geostatistical Analysis
Source: Nutrients. 2021 Jun 19;13(6):2104. doi: 10.3390/nu13062104 (PMC8234472; doi:10.3390/nu13062104)
Supplement: Supplementary file 1 [file nutrients-13-02104-s001.zip › nutrients-1152580-supplementary.pdf]

nutrients

Supplementary Materials

Mapping Geographical Differences and Examining the Determinants of Childhood Stunting in Ethiopia: A Bayesian Geostatistical Analysis

Kedir Y. Ahmed [1,2,*], Kingsley E. Agho [1,3,4], Andrew Page [1], Amit Arora [1,3,5,6,7], Felix Akpojene Ogbo [1,8] and on behalf the Global Maternal and Child Health Research Collaboration (GloMACH) [†]

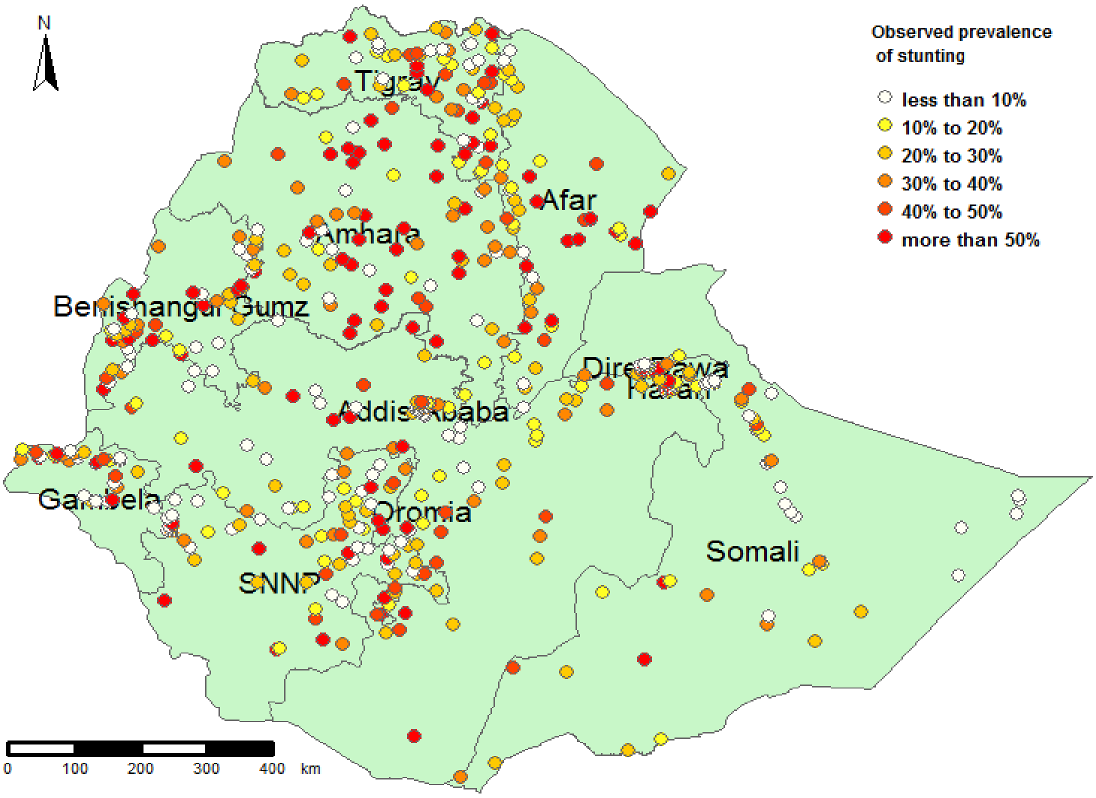

Figure S1. Cluster-level observed prevalence of stunting among children 0–23 months of age in Ethiopia, 2016 EDHS.

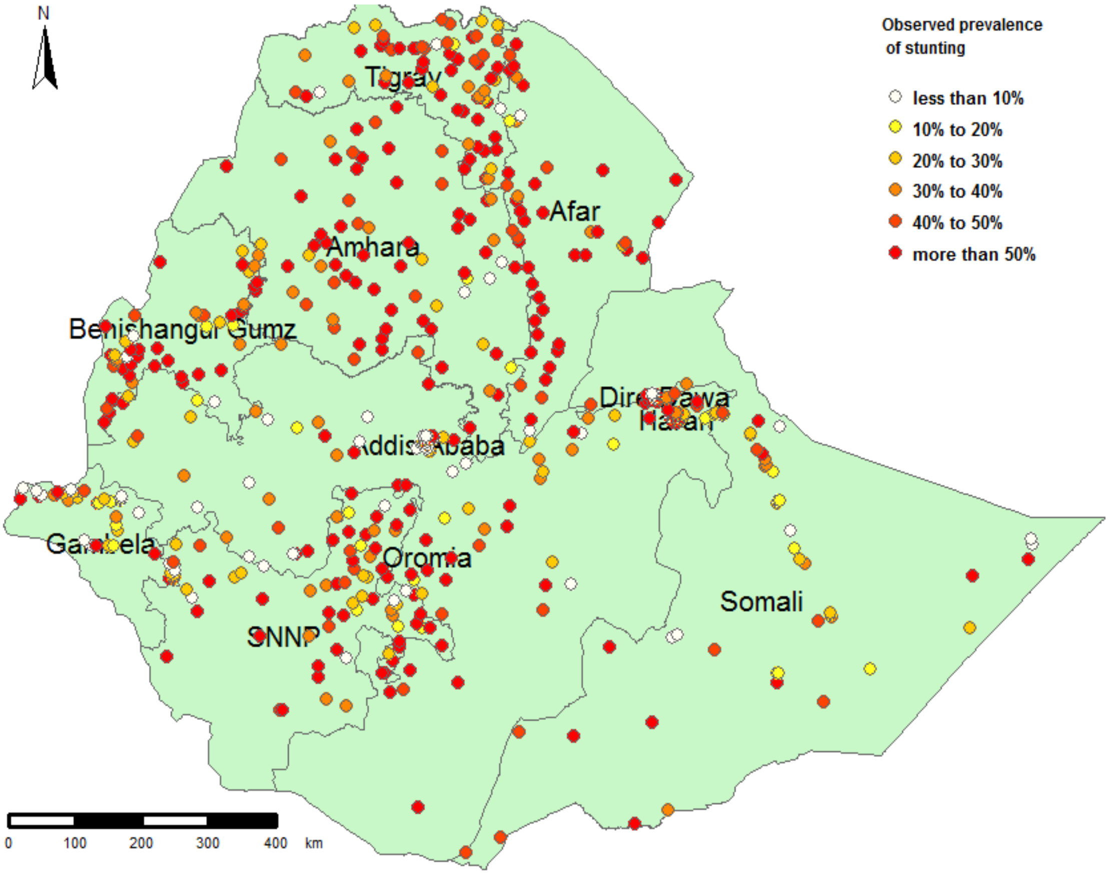

Figure S2. Cluster-level observed prevalence of childhood stunting among children aged 24–59 months in Ethiopia, 2016 EDHS.

Nutrients **2021**, *13*, 2104; doi:10.3390/nu13062104

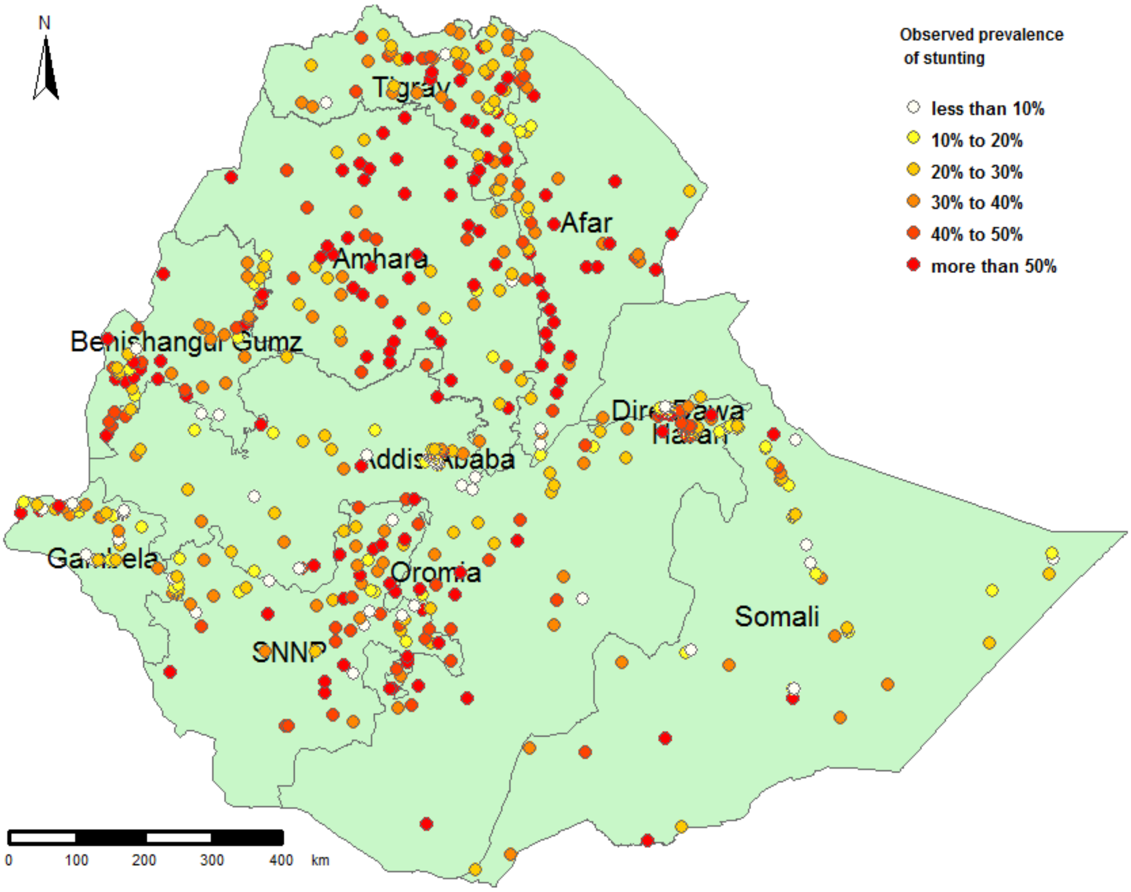

Figure S3. Cluster-level observed prevalence of childhood stunting among children aged 0–59 months in Ethiopia, 2016 EDHS.

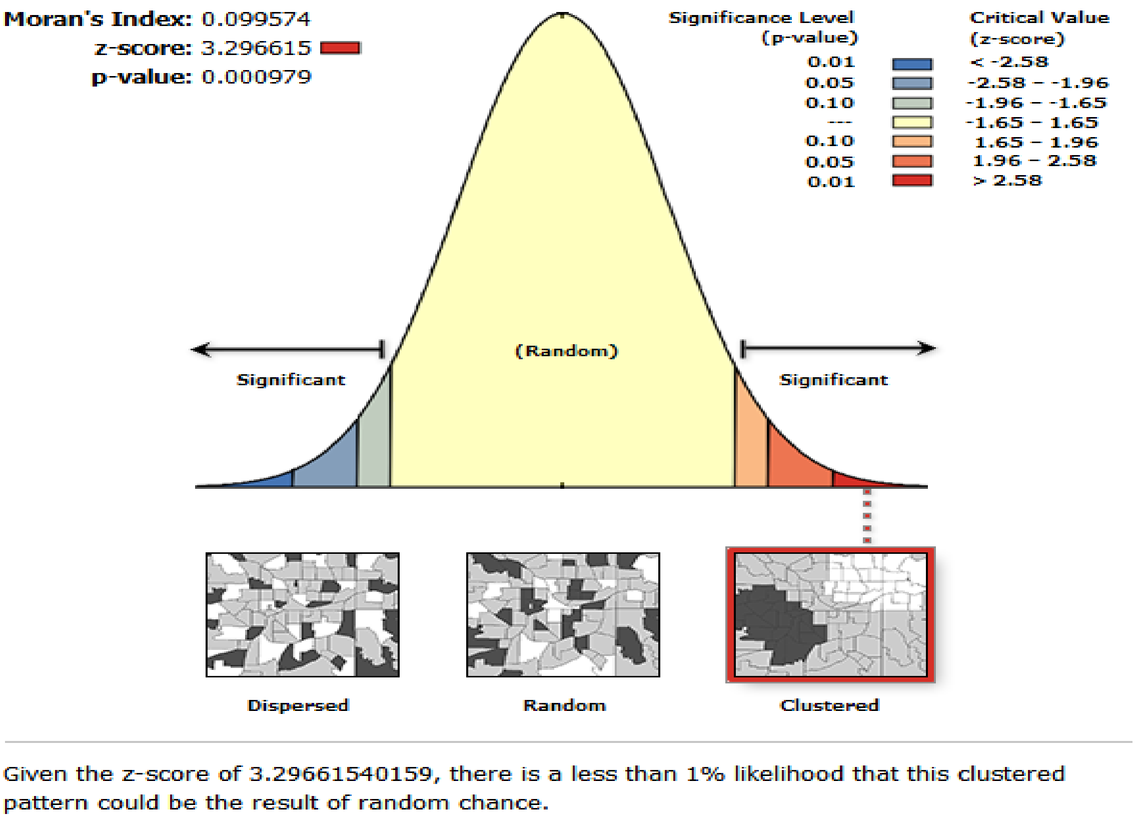

Figure S4. Global spatial autocorrelation analyses for stunting among children aged children 0–59 months.

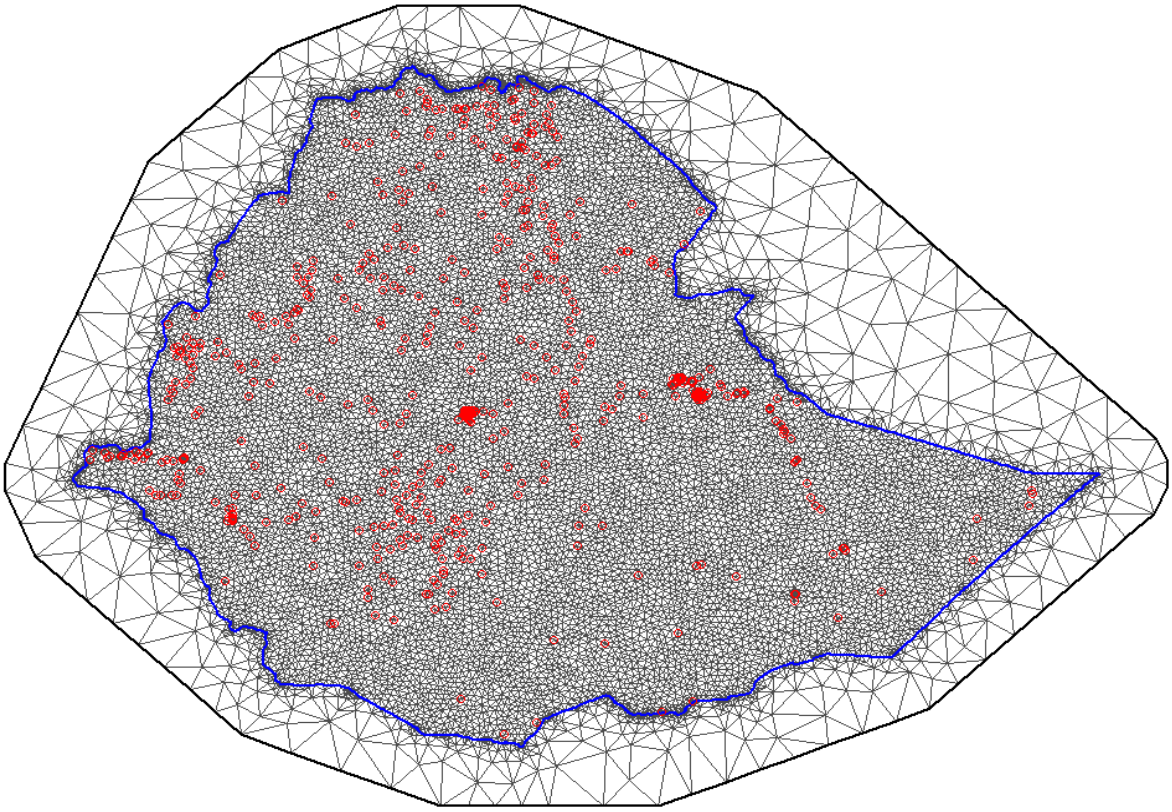

Figure S5. An artificially created mesh to represent the neighbouring structure of the study region in Ethiopia.

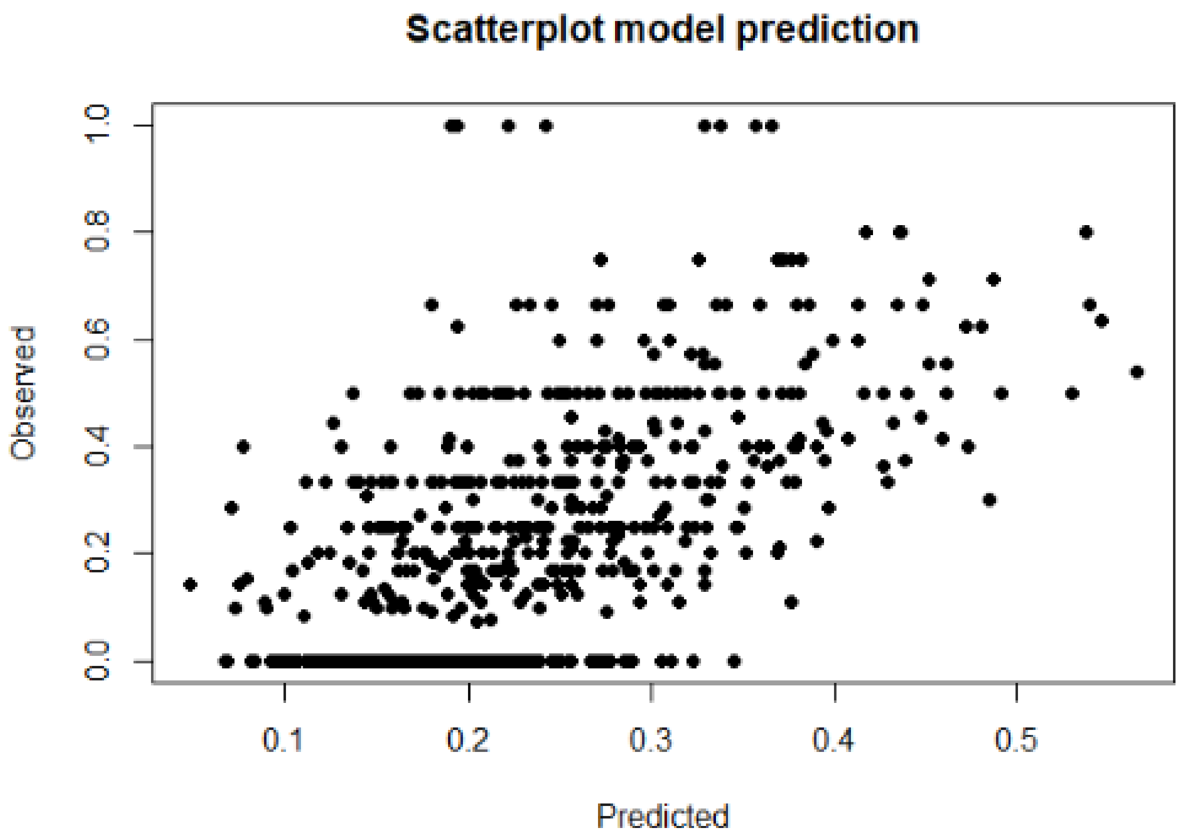

Figure S6. Correlation between observed and predicted values for model validation.

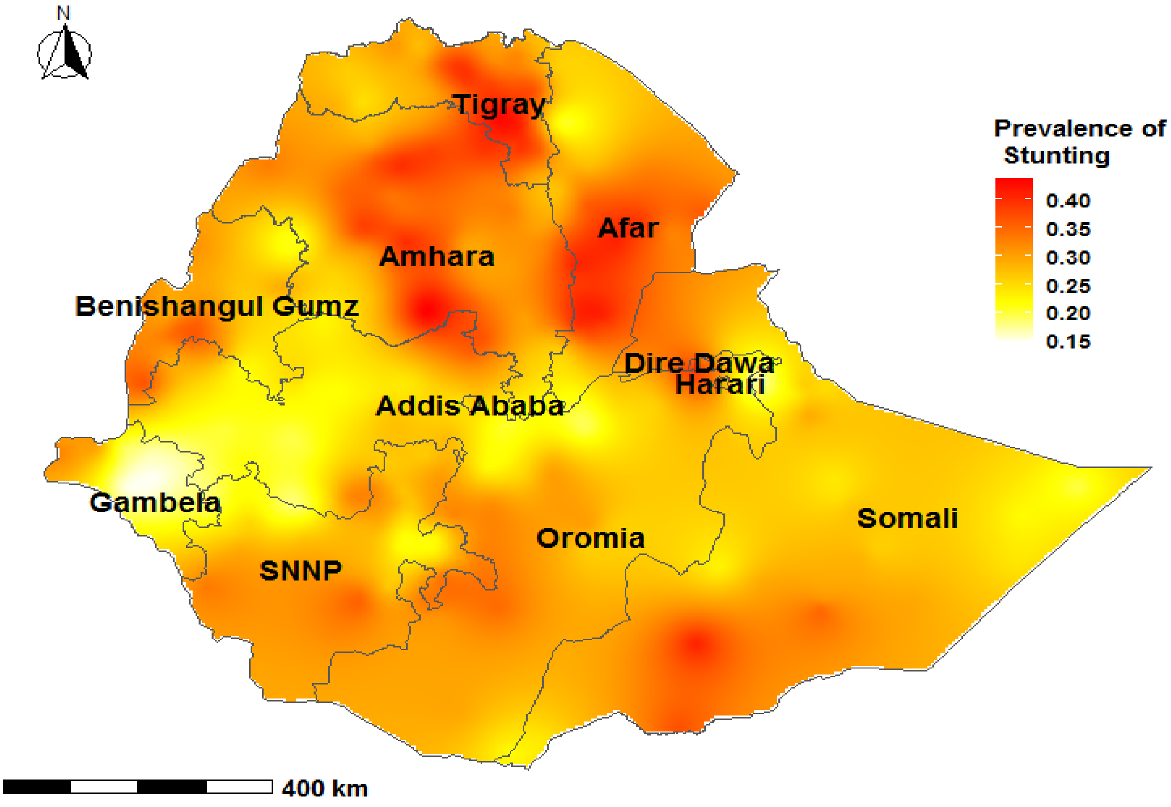

Figure S7. Predicted prevalence of stunting among children 0–59 months of age in Ethiopia, 2016 EDHS.

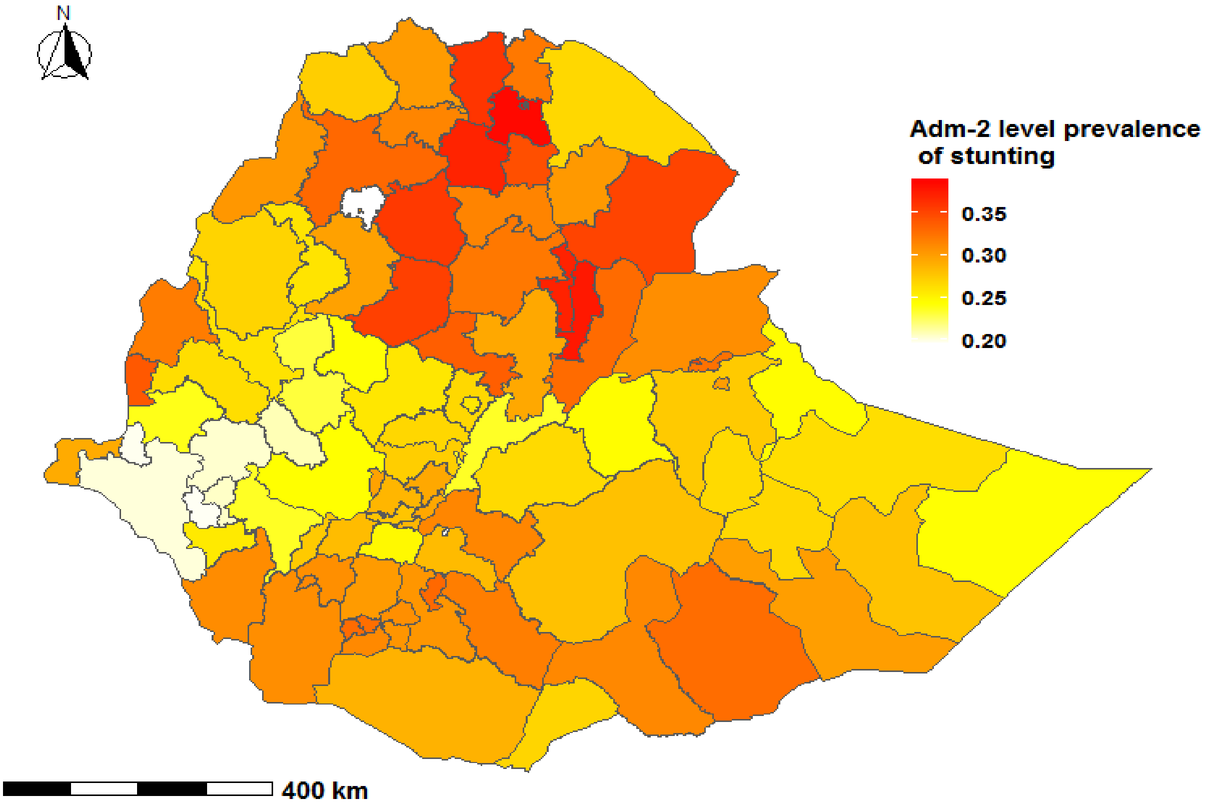

Figure S8. Second administrative level prevalence of stunting among children 0–59 months of age in Ethiopia, EDHS 2016.

Nutrients **2021**, *13*, 2104; doi:10.3390/nu13062104

Table S1. Definition of proximal and contextual factors associated with childhood stunting among children under-five years (identified from a review of conceptual framework and prior studies).

Independent variable	Definitions	Reference category	Data sources
	Proximal factors		
Early initiation of breast-feeding (EIBF) [1]	EIBF was grouped as '1' = 'initiated breastfeeding within 1 h of birth', or '2' = 'Not initiated breast-feeding within 1 h of birth'	Not initiated breastfeed-ing within 1 h of birth	Self-reports
Minimum dietary diver-sity (MDD) [1]	MDD was grouped as '1' = 'met MDD' or '2' = 'did not meet MDD'	Did not meet MDD	Self-reports
Minimum meal fre-quency (MMF) [1]	MMF was grouped as '1' = 'met MMF' or '2' = 'did not meet MMF'	Did not meet MMF	Self-reports
Type of foods in the past 24 hrs [2]	The type of food consumed within the past 24 hours was grouped as '1' = 'only breastmilk', '2' = 'breastmilk and supplementary foods' or '3' = 'no breastmilk'	No breastmilk	Self-reports
Duration of breastfeed-ing [2]	Grouped as '1' = 'less than 12 months' or '2' = '12 months or more'	12 months or more	Self-reports
Bottle feeding [1]	Grouped as '1' = 'bottle feeding' or '2' = 'no bottle feeding'	No bottle feeding	Self-reports
Diarrhoea [3,4]	Diarrhoea was defined as a passing of abnormal stools during the two weeks preceding the survey. Diarrhoea was grouped as '1' = 'experienced diarrhoea' or '2' = 'did not experience diarrhoea'	Did not have diarrhoea'	Self-reports
Acute respiratory infec-tion (ARI) [3,4]	ARI was defined as symptoms of cough and shortness of breath during the two weeks preceding the survey. ARI was grouped as '1' = 'experienced ARI' or '2' = 'did not experience ARI'	No	Self-reports
Anaemia [4,5]	Anaemia was defined as a haemoglobin count of less than 11 grams per decilitre (g/dl). Anaemia was grouped as '1' = 'Anaemic (<11g/dl)' or '2' = 'Not anaemic (>11g/dl)'. In 2016 EDHS, information on haemoglobin count was collected for children 6–59 months.	Not anaemic	Haemoglobin meas-urements
	Proximal contextual factors		
Maternal nutritional sta-tus [6,7]	Maternal nutrition was grouped as '1' = 'underweight (BMI <18.5 kg/m^2)', '2' = 'normal weight (BMI ≥18.5 kg/m2 and BMI ≤24.9 kg/m^2)' or '3' = 'overweight or obese (BMI ≥25.0 kg/m^2)'	Normal weight	Anthropometric measurements
Mothers' and fathers' ed-ucational status [8-10]	Mothers' and fathers' educational status were grouped as '1' = 'no schooling', '2' = 'primary education' or '3' = 'secondary education or higher'	No schooling	Self-reports
Mothers' and father's employment [8–10]	Mothers' and fathers' employment were grouped as '1' = 'no employment', '2' = 'formal employment', or '3' = 'informal employment'	No employment	Self-reports
Maternal age [8–10]	Maternal age was grouped as '1' = '15–24 years', '2' = '25–34 years' or '3' = '35–49 years'.	15–24 years	Self-reports
Household wealth index [8–11]	Household wealth index was grouped as '1' = 'poor', '2' = 'middle', or '3' = 'rich. For this study, 40% of the households was referred to as 'poor households', '20% of them as 'middle-class households', and the top 40% as rich households [2].	Poor	Self-reports
Source of drinking water and type of toilet facility [8–10]	The source of drinking water and type of toilet facility were grouped as '1' = 'improved' or '2' = 'not improved'	Not improved	Self-reports

Type of cooking fuel [8–10]	Type of cooking fuel was grouped as '1' = 'cleaned or '2' = 'not cleaned'	Not cleaned	Self-reports
Frequency of ANC visits [8–10]	Frequency of ANC visits was grouped as '1' = 'none', '2' = '1–4 visits' or '3' = 'four and above visits'	None	Self-reports
Place of birth [8–10]	Place of birth of was grouped as 'home 'or 'health facility birth'	Home	Self-reports
Perceived birth size [8–10]	Perceived birth size was grouped as '1' = 'larger than average', '2' = 'average' or '3' = 'smaller than average', and birth order (categorized as '1', '2–4' or '5 and above').	Larger than average	Self-reports
Child age [1]	Child age was grouped as '1' = 'less than 6 months', '2' = '6–11 months', '3' = '12–17 months', '4' = '18–23 months' or '5' = '24–59 months'	Less than 6 months	Self-reports
	Environmental contextual factors		
Media exposure factors [8–10]	Media exposure factors included listening to radio, reading magazine, and watching television. Media exposure factors were grouped as '1' = 'Yes' or '2' = 'No'	No	Self-reports
Mean annual daytime land surface temperature (DLST) [12,13]	DLST was defined daytime temperature of the land surface (skin temperature), detected by satellites by looking through the atmosphere to the ground [12]. The DLST was grouped as '1' = '<30.0 ^{0}C', '2' = '30–34.9 ^{0}C', or '3' = '+35.0 ^{0}C' [13].	'< 30.0 ^{0}C	Spatial resolutions (6 km by 6 km) obtained from MODIS
Mean annual rainfall [12,14]	Mean annual rainfall was grouped as '1' = 'low' (less than 142 mm), '2' = 'medium' (142–1200 mm) or '3' = 'high' (more than 1200 mm) [58].	Low	Spatial resolutions (5km by 5km)
Aridity [12]	Aridity was defined as the mean annual precipitation divided by mean annual potential evapotranspiration ranged from 0 (most arid) to 300 (most wet) [40]. The aridity index was grouped as '1' = 'arid', aridity index of less than 17.5, '2' = 'semi-arid', aridity index from 17.5 up to 32.5 or '3' = 'wet', aridity index above 32.5	Wet	Spatial resolution for covariates (55 km by 55 km)
The number of wet days per annum [12]	The number of wet days was defined as the mean number of days receiving rainfall within the 2 km (urban) or 10 km (rural) buffer. The number of wet days was grouped as '1' = 'low' (less than six), '2' = 'medium' (from 6 up to 8 wet days), or '3' = 'high' (more than 8 wet days)	Low	Spatial resolution for covariates (55 km by 55 km)

Table S2. Prevalence of stunting by proximal and contextual factors of stunting among children under-five years in Ethiopia, 2016 EDHS [*n* = 9,089].

Variables	Prevalence of stunting		
	**0–23 months of age**	**24–59 months of age**	**0–59 months of age**
	***n* (%)**	***n* (%)**	***n* (%)**
Child feeding factors			
Early initiation of breastfeeding (EIBF)			
No	315 (28.8)	-	-
Yes	767 (27.0)	-	-
Minimum dietary diversity (MDD)			
No	991 (28.0)	-	-
Yes	90 (22.9)	-	-
Minimum meal frequency (MMF)			
No	665 (25.0)	-	-
Yes	417 (32.6)	-	-
Bottle feeding			
No	975 (28.8)	-	-
Yes	107 (19.6)	-	-
Duration of breastfeeding			-
≤12 months	361 (16.8)	-	361 (16.8)
>12 months	720 (40.4)	-	3298 (44.6)
General feeding status (in 24 hours)			
Only breastmilk	124 (17.3)	607 (59.4)	732 (42.0)
Breastmilk + supplements	804 (29.1)	27 (56.3)	832 (29.6)
No breastmilk	153 (33.7)	1943 (42.8)	2096 (42.0)
Child factors			
Mother's perceived baby size at birth			
Larger than average	287 (24.6)	759 (41.4)	1046 (34.9)
Average	424 (25.8)	1090 (44.7)	1513 (37.1)
Smaller than average	371 (33.0)	729 (54.3)	1100 (44.6)
Diarrhoeal diseases			
No	859 (26.1)	2324 (45.6)	3182 (38.0)
Yes	220 (34.4)	254 (49.8)	474 (41.3)
Acute respiratory infection			
No	977 (27.1)	2413 (45.7)	3390 (38.1)
Yes	104 (32.2)	164 (50.3)	269 (41.3)
Childhood anaemia			
No	234 (29.9)	1093 (39.5)	1327 (37.4)
Yes	674 (33.1)	1460 (53.0)	2134 (44.6)
Maternal factors			
Maternal nutritional status			
Normal	761 (26.8)	1957 (46.9)	764 (41.1)
Underweight	284 (35.1)	480 (45.8)	2717 (38.8)
Overweight	21 (10.4)	96 (35.7)	117 (24.9)
Obesity	8 (17.3)	16 (24.8)	24 (21.7)
Maternal educational status			
No schooling	700 (29.6)	1899 (48.8)	2599 (41.5)

Primary education	331 (27.0)	597 (42.7)	928 (35.4)
Secondary or higher education	51 (14.7)	82 (25.3)	132 (19.8)
Maternal employment status			
No employment	644 (27.3)	1404 (46.7)	2047 (38.2)
Formal employment	98 (16.4)	400 (41.9)	498 (32.1)
Informal employment	340 (34.7)	774 (47.0)	1113 (42.4)
	Health service factors		
Antenatal care visits			
None	378 (28.7)	597 (52.9)	975 (39.9)
1–3 visits	361 (29.5)	444 (48.8)	805 (37.7)
+4 visits	288 (22.5)	376 (42.3)	664 (30.6)
Place of birth			
Home	756 (30.5)	2116 (47.1)	2871 (41.2)
Health facility	326 (22.4)	462 (41.3)	787 (30.6)
	Household factors		
Household wealth status			
Poor	572 (32.6)	1385 (51.7)	1957 (44.1)
Middle	209 (24.3)	553 (47.8)	762 (37.8_)
Rich	300 (22.8)	639 (36.0)	939 (30.4)
Source of drinking water			
Not protected	715 (29.0)	1692 (47.5)	2407 (40.0)
Protected	367 (25.0)	885 (43.1)	1252 (35.5)
Toilet system			
Not improved	1009 (28.4)	2395 (47.2)	3404 (39.4)
Improved	73 (19.0)	182 (34.2)	255 (27.9)
	Climatic factors		
Daytime land surface temperature			
<30 ^{0}C	459 (25.9)	1173 (44.1)	1687 (38.0)
30–34.99 ^{0}C	461 (28.0)	1953 (46.1)	1514 (38.6)
+35 ^{0}C	183 (32.2)	1326 (45.3)	570 (41.6)
Annual average rainfall (in mm)			
<141 mm	3 (37.2)	61 (53.5)	7 (41.1)
142–1199 mm	791 (28.2)	3296 (45.3)	2651 (39.0)
≥1200 mm	310 (26.3)	1095 (45.2)	1114 (38.0)
Aridity			
Wet	152 (20.8)	467 (43.6)	629 (33.4)
Semi-arid	588 (29.5)	1808 (44.8)	2034 (41.2)
Arid	362 (28.7)	2177 (46.2)	1108 (38.0)
Number of wet days per year			
Low	189 (28.3)	1794b(45.0)	583 (37.0)
Medium	370 (26.8)	1080 (41.5)	1204 (36.9)
High	544 (28.1)	1578 (48.9)	1984 (40.5)

*n indicates weighted count.

Table S3. Second administrative level prevalence of stunting among under-five children in Ethiopia, EDHS 2016.

	Administrative zones	0–23 months age			24–59 months age			0–59 months of age		
		%	LCrI	UCrI	%	LCrI	UCrI	%	LCrI	UCrI
1.	Afder	30.0	12.9	55.4	48.7	24.8	73.5	36.1	12.6	53.2
2.	Agnewak	28.8	12.3	53.2	33.9	15.8	58.5	28.5	11.2	48.2
3.	Alle	31.4	13.7	57.3	46.8	24.7	70.5	37.7	13.8	55.4
4.	Amaro	30.7	13.4	56.0	47.0	25.5	69.8	35.4	13.6	54.5
5.	Arsi	28.6	12.2	53.0	42.3	22.0	65.8	33.7	11.9	49.9
6.	Asosa	29.8	13.3	53.8	49.1	28.9	69.9	36.5	13.9	50.0
7.	Awi	27.6	11.7	51.8	43.8	23.3	67.0	30.4	11.9	49.3
8.	Bale	29.4	12.6	54.4	44.6	21.9	69.9	33.8	11.9	51.6
9.	Basketo	29.6	12.8	54.6	48.5	25.6	72.5	36.0	12.5	52.2
10.	Bench Maji	28.2	12.1	52.4	43.4	23.3	66.3	34.0	12.4	50.1
11.	Borena	29.5	12.7	54.8	43.5	20.5	69.7	33.8	12.0	52.3
12.	Buno Bedele	27.7	11.6	52.0	36.7	17.0	62.3	29.2	10.8	48.2
13.	Burji	31.3	13.8	56.6	45.3	23.9	68.6	34.4	13.7	53.7
14.	Central	29.4	13.0	53.4	54.0	32.8	74.1	37.7	14.3	51.0
15.	Central Gondar	29.7	12.8	54.7	48.0	25.9	71.1	35.7	13.2	52.8
16.	Daawa	29.1	12.4	54.1	42.0	19.6	68.2	31.9	11.5	51.1
17.	Dawuro	30.2	13.2	55.1	46.8	25.7	69.4	34.4	13.4	52.6
18.	Derashe	33.4	15.0	59.2	46.0	25.0	68.7	38.2	15.8	56.6
19.	Dire Dawa rural	29.6	13.8	52.1	49.2	31.5	67.6	38.3	14.4	46.0
20.	Dire Dawa urban	27.8	12.9	49.6	53.0	35.4	70.2	40.4	13.0	41.2
21.	Doolo	28.5	12.0	53.2	44.1	21.4	69.8	31.7	11.7	51.2
22.	East Gojam	29.6	12.9	54.2	52.6	30.8	73.8	38.4	14.7	54.5
23.	East Hararge	28.7	12.3	53.2	43.2	22.2	67.1	33.3	11.7	49.6
24.	East Shewa	27.6	11.7	51.7	38.2	19.2	61.9	29.8	10.9	47.7
25.	East Wellega	28.4	12.0	52.8	37.7	18.1	62.6	31.1	11.5	49.4
26.	Eastern	27.3	11.9	50.7	50.9	31.2	70.7	35.6	14.4	50.2
27.	Erer	29.0	12.3	54.0	43.1	20.0	69.8	33.6	11.9	52.3
28.	Etang Special woreda	30.0	13.2	54.5	29.4	13.8	52.0	26.2	11.6	47.2
29.	Fafan	28.1	12.0	52.3	40.6	20.9	64.2	31.2	11.4	48.4
30.	Finfine Special	26.6	11.6	49.7	40.2	21.9	61.9	33.1	11.8	46.9
31.	Gamo	30.0	13.2	54.7	47.6	26.8	69.5	36.3	14.5	53.2
32.	Gedeo	33.1	15.6	57.1	52.9	33.4	72.1	42.1	18.7	56.1
33.	Gofa	30.2	13.1	55.3	47.7	25.5	71.2	36.3	13.0	52.7
34.	Guji	30.0	13.0	55.1	49.9	26.9	73.5	37.3	13.2	53.6
35.	Guraghe	28.7	12.7	52.4	41.4	22.6	63.2	32.9	13.5	50.0
36.	Hadiya	28.9	12.8	52.5	48.2	28.6	68.7	34.3	14.5	50.2
37.	Halaba Special	32.0	14.6	56.6	46.5	26.9	67.6	37.2	13.9	50.7
38.	Harari	25.8	12.1	46.3	50.2	33.8	66.9	36.2	13.6	41.0
39.	Horo Gudru Wellega	28.2	11.9	52.6	40.8	20.1	65.6	31.2	11.4	50.1
40.	Ilu Aba Bora	28.5	12.2	52.8	35.7	16.5	61.2	30.1	11.5	49.8
41.	Jarar	28.4	12.0	53.1	42.8	20.7	68.4	32.8	11.6	50.6
42.	Jimma	28.1	12.0	52.3	42.9	22.9	66.0	33.0	12.3	50.1
43.	Kefa	28.1	12.1	52.0	39.5	20.5	62.6	30.7	11.8	48.4
44.	Kelem Wellega	28.2	12.0	52.6	40.0	20.0	64.5	31.9	11.7	50.3
45.	Kemashi	27.7	11.8	51.8	42.8	22.6	66.2	32.6	12.4	50.1
46.	Kembata Tibaro	30.7	13.8	54.8	47.6	28.2	68.1	33.5	13.9	49.2
47.	Konso	31.0	13.5	56.8	44.8	22.8	69.2	35.1	13.2	54.1
48.	Konta Special	29.8	13.0	54.5	44.1	23.4	67.4	34.3	13.3	52.7
49.	Korahe	29.0	12.4	53.8	41.6	19.9	67.2	33.9	12.5	52.4
50.	Liban	29.2	12.5	54.1	49.7	25.5	74.4	35.4	12.8	53.5

51.	Mao Komo Special	28.7	12.5	52.9	51.9	30.1	73.6	41.3	15.9	56.1
52.	Mejenger	25.1	10.4	48.1	37.8	19.9	60.3	28.4	11.8	46.3
53.	Mekele Special	29.4	13.4	52.4	56.6	37.4	74.6	42.7	15.7	49.4
54.	Metekel	28.9	12.5	53.4	43.5	22.6	67.2	31.7	12.6	51.1
55.	Mirab Omo	29.1	12.5	53.8	48.5	25.3	72.8	36.5	12.5	52.5
56.	Nogob	29.2	12.4	54.2	42.5	19.8	69.0	33.3	11.9	52.2
57.	North Gondar	28.9	12.4	53.5	48.1	26.2	70.8	31.6	11.8	49.4
58.	North Shewa	28.5	12.2	52.9	46.3	24.9	69.4	32.7	12.6	51.2
59.	North Shewa	29.6	12.9	54.3	50.1	27.8	72.8	38.3	14.0	54.7
60.	North Wello	29.8	13.1	54.4	46.8	25.4	69.5	32.8	13.7	52.6
61.	North Western	27.2	11.5	51.0	49.2	28.1	71.0	32.3	12.1	48.2
62.	Nuwer	33.2	15.0	58.7	39.9	20.0	63.8	38.9	15.6	55.1
63.	Oromia	29.9	13.1	54.7	55.3	32.7	76.1	40.7	16.5	58.4
64.	Region 14	23.8	10.8	44.0	39.0	23.5	57.3	33.0	12.7	40.1
65.	Shabelle	29.9	12.9	55.1	45.0	22.6	70.0	34.9	12.8	53.1
66.	Sheka	25.8	10.8	49.1	38.2	20.2	60.7	29.1	11.9	47.4
67.	Sidama	30.1	13.5	54.0	44.4	25.9	64.9	33.5	14.1	49.4
68.	Siltie	29.9	13.3	54.1	44.8	25.6	66.0	36.0	14.6	51.8
69.	Siti	29.0	12.4	53.8	45.8	23.0	70.5	33.9	12.0	51.7
70.	South Eastern	32.0	14.5	56.5	53.4	32.9	73.1	41.8	14.8	52.0
71.	South Gondar	29.8	13.0	54.5	50.5	28.4	72.7	37.0	13.5	52.6
72.	South Omo	29.5	12.6	54.7	46.2	22.8	71.7	34.7	12.2	52.6
73.	South Wello	30.3	13.4	55.1	47.3	25.5	70.2	33.4	13.2	51.8
74.	South West Shewa	29.3	12.9	53.4	41.2	21.9	64.3	34.8	13.8	52.9
75.	Southern	30.4	13.6	54.5	49.6	29.3	70.4	37.3	15.2	52.4
76.	Wag Hamra	32.2	14.2	58.2	48.9	26.2	72.1	37.3	13.6	53.6
77.	West Arsi	30.8	13.7	55.5	47.6	26.7	69.6	38.4	14.3	53.4
78.	West Gojam	27.4	11.6	51.4	48.2	27.3	70.0	33.0	12.3	49.4
79.	West Gondar	29.3	12.5	54.4	45.3	22.4	70.5	34.7	12.0	52.0
80.	West Guji	29.9	13.1	54.5	47.6	26.0	70.4	35.2	12.6	51.0
81.	West Hararge	28.7	12.2	53.5	37.3	17.6	62.3	30.7	10.6	47.8
82.	West Shewa	28.6	12.3	52.9	41.4	21.4	64.8	31.8	12.1	49.8
83.	West Wellega	28.0	11.9	52.2	44.3	23.6	67.8	33.6	12.2	50.3
84.	Western	27.6	11.5	52.1	43.6	22.0	68.1	30.5	11.1	48.8
85.	Wolayita	28.1	12.2	51.9	44.0	24.9	65.2	29.4	11.6	45.5
86.	Yem Special	26.6	11.4	49.9	51.9	31.7	72.0	38.1	16.0	53.6
87.	Zone 1 (Awsi Rasu)	30.5	13.6	55.0	50.0	27.8	72.4	37.4	14.1	53.6
88.	Zone 2 (Kilbet Rasu)	28.5	12.2	53.1	42.5	21.1	67.1	31.2	11.9	50.6
89.	Zone 3 (Gabi Rasu)	28.5	12.2	53.1	48.9	26.7	71.6	35.4	13.1	52.3
90.	Zone 4 (Fantana Rasu)	29.8	13.1	54.5	48.8	27.5	70.9	34.0	14.3	53.5
91.	Zone 5 (Hari Rasu)	29.6	13.0	54.0	55.1	33.7	75.0	41.4	18.4	59.2

%: proportion of stunting; LCrI: lower credible interval; UCrI: upper credible interval.

File S1. Model formulation, development and implementation.

Gaussian random fields (GRF)
GRF is defined as a "collection of random variables where the observations occur in a continuous domain, and where every finite collection of random variables has a multivariate normal distribution".

Geostatistical data are assumed to be a partial realization of a random process. Suppose $\boldsymbol{Zi},\ldots,\boldsymbol{Zn}$ are observations of a spatial variable $\boldsymbol{Z}$ at locations $\boldsymbol{i},\ldots,\boldsymbol{n}$.

$$\{\boldsymbol{Z}(\boldsymbol{i}): \boldsymbol{i} \in \boldsymbol{D} \subset \boldsymbol{R}^2\}\ldots\ldots\text{GRF}$$

D is a fixed subset of $\boldsymbol{R}^2$ and the spatial index $\boldsymbol{Zi}$ varies continuously throughout $\boldsymbol{D}$. $\boldsymbol{Zi}$ can observed at a finite set of locations for practical reasons. These properties are helpful for predicting outcomes at unobserved locations, and for the construction of a spatially continuous surface of the outcome of study.

A less restrictive form of the second-order stationarity (or weakly stationarity) employed as a random process. Under this form, the process has constant mean at each location, and the covariance depend only on the distances between locations

$$\boldsymbol{E}[\boldsymbol{Z}(\boldsymbol{s})] = \boldsymbol{\mu}, \forall \boldsymbol{s} \in \boldsymbol{D},$$
$$\boldsymbol{Cov}(\boldsymbol{Z}(\boldsymbol{s}), \boldsymbol{Z}(\boldsymbol{s}+\boldsymbol{h})) = \boldsymbol{C}(\boldsymbol{h}), \forall \boldsymbol{s} \in \boldsymbol{D}, \forall \boldsymbol{h} \in \boldsymbol{R2}$$

At this stage, information related to the continuity and spatial variability can be conveyed using a plot of empirical semi-variogram against the separation distance.

For this study, the Matérn covariance function covariance matrix of GRF, which is used to specify the spatial dependence structure.

The Matérn covariance function is defined as

$$\boldsymbol{Cov}(\boldsymbol{Z}(\boldsymbol{si}), \boldsymbol{Z}(\boldsymbol{sj})) = {\boldsymbol{\sigma}^2}\big/{2^{\nu-1}\boldsymbol{\Gamma}(\boldsymbol{\nu})}\,(\boldsymbol{\kappa}||\boldsymbol{si}-\boldsymbol{sj}||)^{\boldsymbol{\nu}}\boldsymbol{K\nu}(\boldsymbol{\kappa}||\boldsymbol{si}-\boldsymbol{sj}||)$$

$||\boldsymbol{si}-\boldsymbol{sj}||$ denotes the distance between locations $\boldsymbol{si}$ and $\boldsymbol{sj}$, $\boldsymbol{\sigma}^2$ is the variance of the spatial field, and $\boldsymbol{K\nu}(\cdot)$ is the modified Bessel function of second kind and order $\boldsymbol{\nu} > \boldsymbol{0}$. The integer value of $\boldsymbol{\nu}$ determines the mean square variability of the process. $\boldsymbol{K} > \boldsymbol{0}$ is related to the range $\boldsymbol{\rho}$, the distance at which the correlation between two points is approximately zero (correlation decaying point).

Stochastic partial differential equation approach
In geostatistical data, assuming spatially continuous variable underlying the observations that can be modelled using a GRF, and the stochastic partial differential equation approach (SPDE) can be implemented in the R-INLA package to fit a spatial model and predict the variable of interest at un-sampled locations.

Unlike the area data, point data do not have explicit neighbours and thus we would have to calculate the autocorrelation structure between each possible point existing in space. We discretise the study region to create a mesh that would create artificial (but useful) set of neighbours so we could calculate the autocorrelation between points.

The SPDE approximate this by using the Finite Element method to represent the spatial process. In these spatial representation, the spatial domain $\boldsymbol{D}$ (the study region) is divided into a partition of non-intersecting triangles, leading to a triangulated mesh with $\boldsymbol{n}$ nodes and $\boldsymbol{n}$ basis functions.

$$x(s) = \sum_{k=1}^{n} \psi k(s) x k$$

Basis functions $\psi k(\cdot)$ are defined as piecewise linear functions on each triangle that is equal to 1 at vertex k, and equal to 0 at the other vertices.

Model formulation and development

Conditional on the true prevalence of stunting $P(zi)$ at cluster zi, we assume a binomial distribution for Yi. Let Yi be the number of stunted children out of Ni sampled at cluster i:

$$Yi|P(Zi) \sim Binomial(Ni, P(Zi))$$

$$logit(P(Zi)) = B0 + Bxi + \omega i + \varepsilon i$$

$$\omega i \sim GP(0, \Sigma)$$

Here,

- $B0$ denotes the intercept;
- B is the coefficient for explanatory variables;
- ωi is a correlated spatial error term, accounting for spatial autocorrelation between data points; and
- $\varepsilon i \sim N(0, \sigma^2{}_{nugg})$ is an independent error term known as nugget effect.

Model Implementation using R-INLA

1. Loading datasets

```
# loading merged individual level and geo-covariates at cluster level
geo.df.cov <- read_rds("E:/Bayesian/Stunting/Working datasets/geo_df_cov_U2.rds")

# loading administrative shape files
adm0 <- readOGR("E:/Bayesian/Working datasets/eth_admbnda_csa_bofed_20191028_shp/eth_admbnda_adm0_csa_bofed_20190827.shp")
adm1 <- readOGR("E:/Bayesian/Working datasets/eth_admbnda_csa_bofed_20191028_shp/eth_admbnda_adm1_csa_bofed_20190827.shp")
adm2 <- readOGR("E:/Bayesian/Working datasets/eth_admbnda_csa_bofed_20191028_shp/eth_admbnda_adm2_csa_bofed_20190827.shp")

#converting the main dataset to dataframe
geo.df.cov.df <- data.frame(geo.df.cov)

#Retained the outcome variable and covariates after variable selection
finalcov <- dplyr::select(geo.df.cov.df, c(DHSCLUST, LONGNUM, LATNUM, stunting, MDD, MMF, MAD, eibf, bofeed,
                          momwork, childfeed_rec, birthsize_rec, resplace, childage, DurBF, momedu, wealth, BMIC_rec,SWater,
                          toiletsystem, ancvisit, placebirth, anemia, ari, Diarrhea, aridity, ann_rainfall, DLST, wetdays))
#Preparing data for cross validation
aggr <- aggregate(cbind(stunting, pop)~   DHSCLUST, data=finalcov, sum)
aggr$prev <-   aggr$stunting/aggr$pop
set.seed(206)
id.cross <- sample(c(1:nrow(aggr)),ceiling(nrow(aggr)*0.75))
aggr$prev[-id.cross] <- NA
link <- rep(NA,nrow(aggr))
link[which(is.na(aggr$prev))] <- 1
observed.values <- aggr$prev[is.na(link)==F]
```

2. Creating SPDE model

2.1. **Mesh construction**

```
#creating spatial point data frame and assigning coordinate reference system
xy <- finalcov[,c(2,3)]
```

```
my.sf.point <- st_as_sf(x = geo.df.cov, coords = xy, crs = "+proj=longlat +datum=WGS84 +ellps=WGS84 +towgs84=0,0,0")
sps <- as(my.sf.point, "Spatial")
spst <- spTransform(sps, CRS("+proj=longlat +datum=WGS84"))#setting the correct coordinate system

#adding the variables longitude and latitude to the data frames
geo.df.cov[, c("LONGNUM", "LATNUM")] <- coordinates(spst)
points.sf <- st_as_sf(spst) #cluster points as sf file (will be used for mapping in later steps)
bdry.sf <- st_as_sf(adm1) #sf file created from the regional boundaries (will be used for mapping in later steps)

#artificial mesh for spatial autocorrealation##################
coo <- cbind(geo.df.cov$LONGNUM, geo.df.cov$LATNUM)
bdry <- inla.sp2segment(adm0)
bdry$loc <- inla.mesh.map(bdry$loc)

mesh <- inla.mesh.2d(loc = coo, boundary = bdry, max.edge=c(0.05, 1),
                     max.n=c(48000, 16000), ## Safeguard against large meshes.
                     max.n.strict=c(128000, 128000), ## Don't build a huge mesh!
                     cutoff=0.01, ## Filter away adjacent points.
                     offset=c(0.4, 1)) ## Offset for extra boundaries, if needed.
mesh$n
plot(mesh)
points(coo, col = "red")
```

2.2. SPDE and projection matrix A

```
spde <- inla.spde2.matern(mesh = mesh, alpha = 2, groupsconstr = TRUE) #Building SPDE
A.est <- inla.spde.make.A(mesh, loc = coo) #projection matrix
```

2.3. INLA stack

```
###............................the INLA stack for estimation............................##
stk.est.stunt.u2.new <- inla.stack(data=list(y=finalcov$stunting), #the response
                          A=list(A.est, 1, 1),    #the A matrix; the 1 is included to make the list(covariates)
                          effects = list(c(i=1:spde$n.spde, list(Intercept = 1)), # specify the manual intercept!
```

```
list(finalcov[,5:29]),
cluster = finalcov$DHSCLUST),

#this is a quick name so you can call upon easily
tag='est.stunt.u2')
```

2.4. Model fitting

#the non-spatial random intercept model

```
f.stunt.nonsp.u2 <- y ~ relevel(childfeed_rec, 2) + MDD + MMF + MAD + eibf + bofeed + DurBF + momedu + birthsize_rec + wealth + relevel (BMIC_rec, 2) + momwork + SWater + toiletsystem + ancvisit + placebirth + anemia + ari + Diarrhea + relevel(aridity,3) + ann_rainfall + DLST + wetdays + f(cluster, model = "iid")

model_stunt_nonsp_u2<- inla(f.stunt.nonsp.u2, data=inla.stack.data(stk.est.stunt.u2),family= 'binomial',
control.predictor=list(A=inla.stack.A(stk.est.stunt.u2), link = 1, compute=TRUE),
control.compute = list(dic = TRUE, waic = TRUE, config = TRUE),
verbose = FALSE)
```

##refit for best model (including geo-spatial model for predition)

```
f.stunt.u2 <- y ~ relevel(childfeed_rec, 2) + MDD + MMF + MAD + eibf + bofeed + DurBF + momedu + birthsize_rec + wealth + relevel (BMIC_rec, 2) + momwork + SWater + toiletsystem + ancvisit + placebirth + anemia + ari + Diarrhea + relevel(aridity,3) + ann_rainfall + DLST + wetdays + f(cluster, model = "iid", group = cluster, control.group = list(model = "iid")) + f(i, model=spde)

model.stunt.u2 <- inla(f.stunt.u2, data=inla.stack.data(stk.est.stunt.u2,spde=spde),family= 'binomial',
control.predictor=list(A=inla.stack.A(stk.est.stunt.u2), link = 1, compute=TRUE),
control.compute = list(dic = TRUE, waic = TRUE, config = TRUE),
verbose = FALSE)
```

2.5. Model validation

```
fitted.values.logit <- model.stunt.u2$summary.fitted.values[is.na(link)==F,1]
fiited.values <- exp(fitted.values.logit)/(1 + exp(fitted.values.logit))

rmse <- sqrt(mean((observed.values-fitted.values)^2))
```

```
fit0025 <- model.stunt.u2$summary.fitted.values[is.na(link)==F,"0.025quant"]
fit0975 <- model.stunt.u2$summary.fitted.values[is.na(link)==F,"0.975quant"]
coverage <- mean((observed.values>=fit0025)&(observ.val<=fit0975))
```

3. Model prediction at fine spatial grid

```
##..........................................5km by 5km grid for prediction............#
#Projection of the random field
points.em <- mesh$loc
stepsize <- 5 * 1 / 111                      # This is the coordinates units
x.range <- diff(range(points.em[,1]))    # calculate the length of the x range
y.range <- diff(range(points.em[,2])) # calculate the length of the y range
nxy <- round(c(x.range, y.range)/stepsize)    # Calculate the number of cells in the x and y ranges

##................................... Project the spatial field on the mesh vertices....................##
projgrid <- inla.mesh.projector(mesh,
                    xlim = range(points.em[,1]),
                    ylim = range(points.em[,2]),
                    dims = nxy)

#........removing points outside the boundary.........##
xy.in <- inout(projgrid$lattice$loc, bdry$loc)

##.............................prediction location coordinates...........................##
prdcoo <- projgrid$lattice$loc[which(xy.in), ]

#extracted covariates at prediction locations
predcov <- read_rds("E:/Bayesian/Stunting/Working datasets/pred_stunt_cov_U2.rds")

#.......................prediction stack for response variable..................................#
stk.pred.stunt.response.u2 <- inla.stack(data = list(y = NA),
                    A = list(projgrid$proj$A, 1, 1),
                    effects = list(i=1:spde$n.spde, list(Intercept = rep(1, 377 * 298)),
```

```
                list(predcov [,5:14]))
                        tag = 'pred.stunt.response.u2')

#We join all together the three linear predictors
join.stack.stunt.response.u2 <- inla.stack(stk.est.stunt.u2, stk.pred.stunt.response.u2)

#fitting the prediction model
f.pred.stunt.u2 <- y ~ 1 + momedu_pred + SWater_pred + toiletsystem_pred +    ancvisit4_pred + placebirth_pred + aridity_pred + ann_rainfall_pred + DLST_pred + wetdays_pred
+ f(i, model=spde)

model.pred.stunt.response.u2 <- inla(f.pred.stunt.u2, data = inla.stack.data(join.stack.stunt.response.u2), family = "binomial",
                        control.predictor = list(A = inla.stack.A(join.stack.stunt.response.u2), compute = TRUE),
                        control.results=list(return.marginals.random=FALSE, return.marginals.predictor=FALSE))

# prevalence at prediction locations
p.prev.stunt.resp.logit <- model.pred.stunt.response.u2$summary.fitted.values[index.pred.stunt.response.u2,"mean"]
p.prev.stunt.response <- exp(p.prev.stunt.resp.logit)/(1 + exp(p.prev.stunt.resp.logit))
summary(p.prev.stunt.response)
p.prev.stunt.resp <- data.frame(x = prdcoo[, 1], y = prdcoo[, 2],
                        value = p.prev.stunt.response[xy.in], variable = "Predicted prevalence of stunting")
p.prev.stunt.resp.sp = SpatialPixelsDataFrame(points = p.prev.stunt.resp[c("x", "y")], data = p.prev.stunt.resp,
                                proj4string = CRS("+proj=longlat +datum=WGS84"))
p.prev.stunt.resp.r <- as(p.prev.stunt.resp.sp, "RasterStack")
p.prev.stunt.resp.ra <- disaggregate(p.prev.stunt.resp.r$value, 5)
p.prev.stunt.resp.raster <- focal(p.prev.stunt.resp.ra, w=matrix(1,5,5), mean)

#modelled surface map for prevalence
coords_stunt_u2 <- xyFromCell(p.prev.stunt.resp.raster, seq_len(ncell(p.prev.stunt.resp.raster)))
p_prev_stunt_u2_df <- stack(as.data.frame(getValues(p.prev.stunt.resp.raster)))
names(p_prev_stunt_u2_df) <- c('value', 'variable')
prev_stunt_u2_cbind <- cbind(coords_stunt_u2, p_prev_stunt_u2_df)
regions_sf <- cbind(bdry.sf.adm1, st_coordinates(st_centroid(bdry.sf.adm1)))
```

```
#visualizing surface map of stunting
map.stunt_u2 <- ggplot(regions_sf) +
  geom_tile(data = prev_stunt_u2_cbind, aes(x=x, y=y, fill = value), na.rm = T) +
  geom_sf(fill = NA, inherit.aes = F) +
  geom_sf_text(aes(label = ADM1_EN),size = 5, fontface = "bold", colour = "black")+
  scale_fill_gradientn(name = "Prevalence of \n Stunting", labels = scales::comma, colors = col, na.value="white") +
  theme_bw() + theme(axis.text = element_blank(), axis.ticks = element_blank(),
                     axis.line = element_blank(), axis.title.x=element_blank(),
                     axis.title.y=element_blank(), panel.grid.major = element_blank(),
                     panel.grid.minor = element_blank(), panel.border = element_blank(),
                     panel.background = element_blank(), legend.position = c(0.90, 0.7),
                     text = element_text(size = 13, face = "bold")) +
  annotation_scale(location = "bl", width_hint = 0.25, text_cex = 1, text_face = "bold") +
  annotation_north_arrow(location = "tl", which_north = "true",
                         pad_x = unit(0.5, "cm"), pad_y = unit(0.5, "cm"),
                         style = north_arrow_fancy_orienteering)
```

References

1. WHO and UNICEF, *Indicators for assessing infant and young child feeding practices Part 1 Definitions*. 2008.
2. Akombi, B.J., et al., *Stunting and severe stunting among children under-5 years in Nigeria: A multilevel analysis.* BMC Pediatrics, 2017. **17**(1): p. 15.
3. WHO. *Diarrhoeal disease*. 2017. Available online: https://www.who.int/news-room/fact-sheets/detail/diarrhoeal-disease (accessed on 25 November 2020).
4. Croft, et al., *Guide to DHS Statistics: DHS-7*. 2018: Rockville, Maryland, USA.
5. Central Statistics Agency (CSA) [Ethiopia] and ICF International, *Ethiopia demographic and health survey 2016*. 2016, Central Statistical Agency (CSA) and ICF International: Addis Ababa, Ethiopia and Rockville, Maryland, USA.
6. Ahmed, K.Y., et al., *Trends and determinants of underweight and overweight/obesity among urban Ethiopian women from 2000 to 2016.* BMC Public Health, 2020. **20**(1): p. 1276.
7. Ahmed, K.Y., et al., *Factors associated with underweight, overweight, and obesity in reproductive age Tanzanian women.* PLOS ONE, 2020. **15**(8): p. e0237720.
8. Ahmed, K.Y., et al., *Trends and factors associated with complementary feeding practices in Ethiopia from 2005 to 2016.* **n/a**(n/a): p. e12926.
9. Ahmed, K.Y., et al., *Trends and determinants of early initiation of breastfeeding and exclusive breastfeeding in Ethiopia from 2000 to 2016.* International Breastfeeding Journal, 2019. **14**(1): p. 40.
10. Ahmed, K.Y., et al., *Associations between infant and young child feeding practices and acute respiratory infection and diarrhoea in Ethiopia: A propensity score matching approach.* PLOS ONE, 2020. **15**(4): p. e0230978.
11. Ogbo, F.A., P. Ogeleka, and A.O. Awosemo, *Trends and determinants of complementary feeding practices in Tanzania, 2004-2016.* Trop Med Health, 2018. **46**: p. 40.
12. Benjamin Mayala, et al., *The DHS program geospatial covariate datasets manual (second edition).* 2018, ICF: Rockville, Maryland, USA.
13. Tusting, L.S., et al., *Environmental temperature and growth faltering in African children: a cross-sectional study.* The Lancet Planetary Health, 2020. **4**(3): p. e116-e123.
14. Kidanewold, B., Y. Seleshi, and A. Melesse, *Surface Water and Groundwater Resources of Ethiopia: Potentials and Challenges of Water Resources Development*. 2014. p. 97-118.
